# Optimizing just-in-time adaptive interventions for interpersonal distress: mechanisms, prediction, and the challenge of engagement

**DOI:** 10.1038/s41598-026-39518-z

**Published:** 2026-02-11

**Authors:** Agata Jaremba, Sarah O’Reilly, Liam Mason, Tobias Nolte, Madiha Shaikh, Ciarán O’Driscoll

**Affiliations:** 1 Research Department of Clinical, Educational, and Health Psychology, University College London, Gower Street, London, WC1E 6BT UK; 2 Anna Freud, London, UK; 3 Clinical Health Psychology, North East London NHS Foundation Trust, Rainham, UK

**Keywords:** Dynamic network analysis, Expressed Emotion, Just-in-time adaptive interventions, Mindfulness, Mentalization, Health care, Psychology, Psychology

## Abstract

Common mental health disorders (CMD) feature fluctuating emotional and interpersonal symptoms inadequately addressed by traditional weekly therapies. Ecological momentary interventions offer potential for timely support, yet their mechanisms and optimal delivery contexts remain unclear. This secondary analysis of a randomized trial (*N* = 77) compared mindfulness and mentalization micro-interventions triggered by personalized symptom thresholds. We examined dynamic symptom networks, proximal effectiveness, engagement predictors, and distress forecasting in adults with CMD. Dynamic networks revealed stable communities (interpersonal threat, social connection, affective states) with mood as a key bridge. No significant proximal intervention effects were observed. Non-engagement was significantly predicted by high stress (OR = 1.21), elevated mood (OR = 1.22), and perceived criticism (OR = 1.22). Conversely, cumulative symptom triggers (OR = 0.69) and social contact (OR = 0.83) facilitated engagement. The dynamic prediction model achieved fair performance (AUC = 0.66) for next-beep distress. Beyond autoregressive effects, perceived criticism (OR: 1.12) and paradoxically perceived support predicted future distress (OR = 1.14), while warmth was protective (OR = 0.87). Micro-interventions operate through stable networks and may yield cumulative rather than immediate benefits. High stress and criticism impede intervention use despite high need highlighting the necessity for context-sensitive, low-friction adaptive designs to align clinical need with receptivity.

**Supplementary Information:**

The online version contains supplementary material available at 10.1038/s41598-026-39518-z.

## Introduction

Depression and anxiety are the most common mental health disorders (CMD) globally, contributing more to disability than any other illness^1^. Psychological therapy is the recommended first-line treatment^2,3^ with promising outcomes^4^. However, many people do not benefit, and the treatment efficacy may be overestimated^5,6^. Interpersonal distress plays an important role in CMD and is associated with worse treatment outcomes^7,8^. Such factors are not routinely measured in clinical practice^9^, and current treatments often overlook interpersonal functioning^10,11^.

Investigating how and when changes occur can help us understand the intervention process^12,13^. Symptoms are not static; they influence one another, and while interventions may share underlying processes, they often exert differential effects on specific symptoms^14–16^. Ecological momentary assessment (EMA)^17^ allows fluctuations in mood and behaviour to be monitored in daily life via digital technology^18^. This is particularly relevant for capturing responses to environmental triggers, such as social interactions^19^ and affect-event dynamics^20^. Furthermore ecological momentary interventions (EMI) enable microinterventions to be adjusted and delivered in real time in response to an individual’s changing context, known as just in time adaptive interventions (JITAI)^21,22^. Not only could EMIs enable more accessible, timely and personalised treatment, they can also help us better understand how treatments work. For instance, tracking symptoms can help identify which proximal outcomes appear to be influenced by specific microinterventions, elucidating mechanisms of change^23–25^. However, research in this area is nascent, and little is known about optimal EMI design regarding timing, intensity and duration^26^.

This secondary analysis examined change processes and intervention optimization in a feasibility randomized controlled trial comparing mindfulness and mentalization micro-interventions for interpersonal distress in people with CMDs^27^. The primary trial demonstrated high acceptability and adherence, with both interventions producing significant symptom reduction (moderate-to-large effect sizes) and no significant between-group differences. However, mindfulness showed broader momentary effects across interpersonal and emotional domains in EMA items. Two optimization challenges emerged requiring further investigation: (1) the intervention trigger threshold appeared overly sensitive, reducing engagement; and (2) while clinically efficacious, the specific mechanisms of change and symptom responsiveness remained unclear.

This study addresses these gaps through four complementary aims:

**Aim 1** modeled relationships between key interpersonal and affective features associated with the two trial arms, as they changed over time, using dynamic network analysis to determine whether co-fluctuation patterns differed between interventions.

**Aim 2** examined which symptoms improved most following successful intervention delivery, revealing proximal intervention effects.

**Aim 3** identified predictors of intervention non-engagement, highlighting barriers to real-time intervention utilization.

**Aim 4** developed a predictive model to forecast imminent distress, informing precise intervention triggering.

Together, these analyses provide mechanistic insights into how mindfulness and mentalization micro-interventions produce change while identifying actionable strategies for optimizing JITAI delivery efficiency and engagement.

## Methods

### Design

This study was a secondary analysis of a randomized controlled feasibility trial examining JITAIs for interpersonal distress. Data were collected via the mPath app^28^. Full protocol details are available in the primary publication^27^.

### Participants

Data collection occurred between March 2024 and February 2025. Participants were recruited via convenience sampling from a university setting. A total of 84 participants were randomized. Although nine participants disengaged, two provided sufficient data for inclusion in the dynamic network analysis, resulting in a final analytical sample of *N* = 77. Participants ranged in age from 18 to 61 years (M = 23.50, SD = 6.56).

The study was approved by the UCL Ethics Review Committee (Project ID: 26261/001).

### Procedure

#### Inclusion/Exclusion criteria

Participants completed a set of screening questionnaires, which included the Patient Health Questionnaire-9 (PHQ-9)^29^ and the Generalized Anxiety Disorder 7-item scale (GAD-7)^30^. They were invited to proceed if they met the inclusion criteria: (1) Scored above the clinical threshold on either the PHQ-9 (≥ 10) or GAD-7 (≥ 8), or both, (2) Not reporting risk (PHQ-9 item 9, score = 0), (3) Not currently in therapy, (4) Not taking prescribed medication for mental health, (5) Registered with a GP in the UK.

Eligible participants downloaded the mPath app, received a video tutorial on using the app, and were randomly assigned to the mindfulness or mentalization condition.

### EMA/EMI protocol

Participants received four daily notifications scheduled around sleep/wake times. Reminder emails were sent if response rates dropped below 80%.

#### EMA items

(supplementary table S1) assessed dimensions of Expressed Emotion^31^ and captured real-time perceptions of threat and safety known to predict clinical outcomes (criticism, hostility, overinvolvement, support, warmth), affective states (mood and stress) to monitor the immediate affective impact of triggers; and the downstream consequences of distress that often maintains depressive affect^32^ (engagement, interaction, assertiveness).

#### Baseline phase (Days 1–5)

Responses established a baseline across ten variables.

**Intervention Phase (Days 6–28)**: Following the baseline, an intervention was triggered if a response score exceeded 1 SD above the participant’s rolling average on: Low Mood, Criticism, Hostility, Overinvolvement or Support.

#### Micro-interventions

Upon triggering, participants received a validating statement. If they opted to proceed.

- *Mentalization Condition*: Received four prompts focusing on self-perspective, other-perspective, appraising intentions, and alternative responses (integrating self-other domains).
- *Mindfulness Condition*: Received three prompts facilitating a “three-minute breathing space” (Awareness, Gathering, Expanding)^33^.

### Data analysis

Data analyses and visualisation were completed in R (Version 4.0.3) with the following key packages: glmmTMB^34^, pROC^35^ and DynEGA^36^. We employed α = 0.05 for statistical significance while emphasizing effect size interpretation and confidence interval estimation. Cohen’s d interpretation: small (0.2), medium (0.5), large (0.8). Odds ratio interpretation: small (OR = 1.2), medium (OR = 2.0), large (OR = 4.0).

The sample consisted of 77 participants. Within the study, 84 participants in total were randomised to each group. Nine disengaged of whom two had sufficient data to be included in the dynamic network analysis. While simulations recommend ≥ 50 time points per individual, the analysis has been successfully applied with fewer observations^37^.

### Descriptive statistics

We computed standard descriptive statistics (mean, median, SD, skewness) and the Root Mean Square of Successive Differences (RMSSD) to quantify temporal variability. Individual-level metrics were aggregated to produce group-level estimates (e.g., iMedian).

### Aim 1 dynamic network analysis

To model the temporal dynamics of symptom change, we employed Dynamic Exploratory Graph Analysis (DynEGA)^38^. We selected DynEGA specifically because, unlike traditional time-series models, it does not assume stationarity, allowing it to accurately model symptom dynamics that may evolve or fluctuate throughout the intervention period. This analysis provided a foundational understanding of symptom relationships, revealing the extent to which variables change together over time.

The analysis was conducted separately for each condition using both first (D1) and second (D2) derivatives. D1 captures the rate of change (velocity), while D2 reflects change in rate of change over time (acceleration). The first 20 time points (baseline phase) were excluded to focus on intervention-specific dynamics.

Network plots were generated to visualise edges between nodes and community structures. Several network metrics were computed: (1) Pearson correlations between edge weight matrices assessed similarity between networks; (2) within-community edge weights (partial correlations) reflected direct associations between nodes while controlling for all others in the network; (3) bridge strength centrality defined as the sum of a node’s edge weights linking it to nodes in other communities; (4) strength centrality, defined as the sum of absolute edge weights per node, indicated variable influence within the overall network.

Networks were visually compared between conditions to identify structural differences in symptom co-fluctuation. Edges represent regularized partial correlations (Gaussian Graphical Model estimated via GLASSO, γ = 0.50), where higher absolute values indicate stronger unique associations between nodes after controlling for all others.

To assess for potential item confounding we assessed within-person correlations between items (supplementary figure S1). These ranged from *r* = 0 (overinvolvement and mood) to 0.77 (support and warmth), the mean absolute correlation across all pairs was modest (*r* = 0.28). No items reached the rule of thumb criterion of *r* > 0.80^39^. Combining Warmth and Support was considered but retained as separate items, as they capture theoretically distinct facets of positive EE (instrumental support vs. emotional warmth), and the DynEGA analyses show differential associations (rates of change) with other EMA items.

### Aim 2 proximal intervention effectiveness

To identify which symptoms improved most immediately following successful intervention delivery, we assessed proximal intervention effects using Generalized Linear Mixed Models (GLMM). The analysis compared symptom levels at the intervention trigger beep (t_0) to levels at the subsequent measurement occasion (t_1).

Data Processing: To ensure valid proximal comparisons, we excluded instances where the subsequent beep occurred on the following day (overnight gaps). To facilitate effect size interpretation within the model, all outcome variables were standardized (z-scored) across the dataset prior to analysis.

Statistical Model: We fitted separate linear mixed models for each outcome variable within each intervention arm. The model predicted the standardized symptom score based on a binary time indicator (0 = Trigger, 1 = Subsequent Beep), with a random intercept for participants to account for the nested data structure (multiple intervention episodes per participant).

The model specification was:

$${\mathrm{Y}_{zij}}={\beta _0}+{\beta _1}({\mathrm{Tim}}{{\mathrm{e}}_{ij}})+{\mathrm{u}_{0j}}+{\epsilon _{ij}}$$

Where $Y_{zij}$ is the standardized outcome for observation i in participant j, and $\beta_1$ represents the standardized mean difference (analogous to Cohen’s d) between the trigger moment and the post-intervention assessment. Significance was assessed via Wald z-tests ($\alpha$ = 0.05).

### Aim 3 predictors of intervention Non-Engagement

We utilized mixed-effects logistic regression to identify predictors of intervention non-engagement (ignoring the prompt). Predictors included all EMA variables, temporal factors (beep number, weekend), and context (social contact, cumulative intervention count).

### Aim 4 dynamic prediction

We developed a model to forecast imminent distress (at time t + 1) based on current symptom profiles.

#### Outcome

A composite “Distress” variable was created via Principal Component Analysis (PCA) of standardized Mood and Stress variables. “High Distress” was defined as the bottom quartile (< 25th percentile) of the individual’s PC1 distribution.

A Generalized Linear Mixed Model (GLMM) with a binomial distribution (logit link) was fitted to predict the probability of high distress at the subsequent assessment (t + 1). To test whether interpersonal and contextual factors could predict future distress beyond current state affect, we included current Mood and Stress (at time t) as control variables.

The model specified fixed effects for interpersonal perceptions (Criticism, Hostility, Overinvolvement, Support, Warmth), context (social contact, recent intervention engagement), and trigger load (cumulative count of breached thresholds). Temporal controls included time of day, study day and weekend status. A random intercept was included for each participant to account for between-person heterogeneity and the nested nature of the data.

Model specification:

$$\begin{aligned} {\mathrm{Logit}}\left({{\mathrm{p}}_{({\mathrm{i}},{\mathrm{t}}+1)}}\right) = \beta_0 + u_{0{\mathrm{i}}}+\beta_1\:\:{\mathrm{Mood}}_{\mathrm{ti}}+\beta_2\:\:{\mathrm{Stress}}_{\mathrm{ti}}({{\text{State Controls}}}) +\beta_{\mathrm{3}}\:\:{\mathrm{Crit}}_{\mathrm{ti}}+ \\ \beta_{\mathrm{4}}\:\:{\mathrm{Host}}_{\mathrm{ti}}+{\beta_{{5}}\:\:{\mathrm{OverInv}_{\mathrm{ti}} }}+\beta_{{6}}\:\:{\mathrm{Supp}_{\mathrm{ti}}}+\beta_{{7}}\:\:{\mathrm{Warmth}}_{\mathrm{ti}}\left( {{\mathrm{Interpersonal}}} \right){\text{ }}+\beta_{{8}}\:\:{\mathrm{Triggers}}_{\mathrm{ti}}+ \\ \beta_{{9}}\:\:{\mathrm{Contact}}_{\mathrm{ti}}+\beta_{{10}}{\mathrm{RecInt}}_{\mathrm{ti}}\left( {{\mathrm{Context}}} \right) + \beta_{{11}}{\mathrm{Time}}_{\mathrm{ti}}+\beta_{{12}}\:\:{\mathrm{Day}}_{\mathrm{ti}}+\beta_{{13}}\:\:{\mathrm{Weekend}}_{\mathrm{ti}}\left( {{\mathrm{Temporal}}} \right) \end{aligned}$$

#### Validation

Model performance was assessed via AUC (Area Under the Curve) using DeLong’s method for confidence intervals.

## Results

### Descriptive statistics

The mean age of participants was 23.8 (SD = 6.75), with a median age of 22. Most of the participants identified as female (83.10%). Most participants were Asian or Asian British (61%), followed by White (23.4%), Other ethnic group (7.8%), Mixed or multiple ethnic groups (5.2%), and Black, Black British, Caribbean or African (2.6%). In terms of employment status, most participants were full-time students (61.0%), followed by those in full-time employment (18.2%), studying while employed part or full-time (13.0%), part-time employed (3.9%), and unemployed (3.9%).

**Fig. 1:**
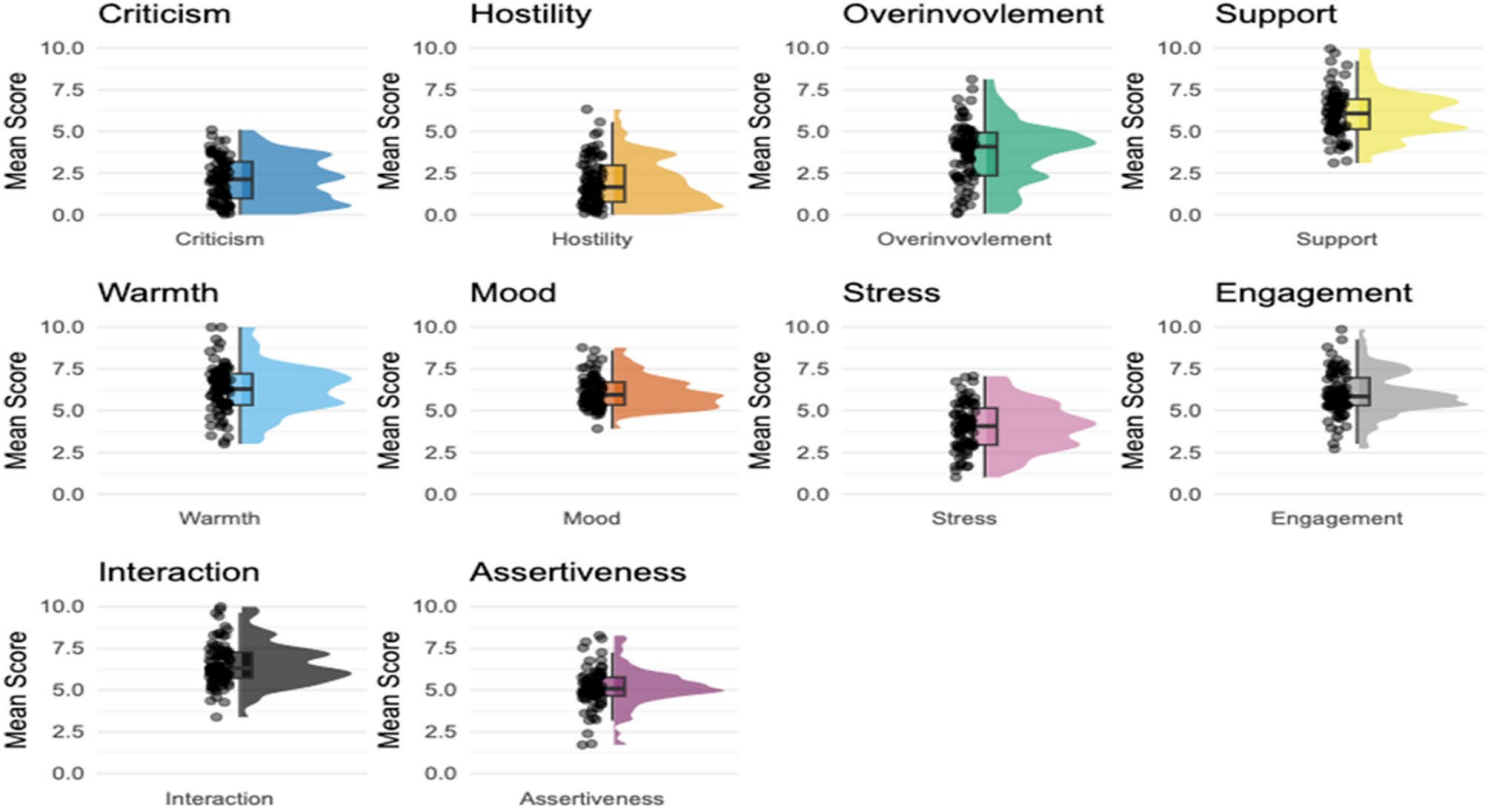
Raincloud Plots of Mean Scores for EMA Variables. Note. The raincloud plots visually represent the distribution of mean scores (iMean) across participants for each EMA variable. Each plot includes a half-violin plot showing the shape of the distribution, a box plot displaying the median interquartile range, overall spread, and jittered raw data points that illustrate the density of responses and highlight outliers. The whiskers on the boxplot extend to 1.5 times the interquartile range.

On average, participants reported lower levels of Criticism, Hostility, and Overinvolvement in their interactions compared to Warmth (Fig. 1). Participants also reported high levels of Engagement, Interaction and Mood, suggesting generally positive interpersonal experiences. The iRMSSD values were above 2 for most items, indicating high within-person fluctuations over time (Table 1). This suggests that although some variables were rated as consistently high or low, individuals still experienced considerable variability from moment to moment. Between-person reliability (RKF) estimated over the baseline phase was excellent (RKF > 0.88), indicating a highly stable estimate of individual differences across items. Reliability of change estimates (RC) ranged from 0.64 to 0.75 demonstrating a high ability to detect momentary fluctuations, consistent with or exceeding typical benchmarks for single-item EMA measures^40^.

**Table 1:** Descriptive statistics for the EMA items.

Trait	Mean	Median	SD	Skew	RMSSD	*R*_KF_​	*R*_C_​
Criticism	2.12 (1.32)	1.61 (1.54)	1.81 (0.70)	1.73 (1.57)	2.22 (0.90)	0.89	0.71
Hostility	1.98 (1.43)	1.49 (1.67)	1.78 (0.74)	1.87 (1.77)	2.20 (0.90)	0.91	0.68
Overinvolvement	3.72 (1.80)	3.41 (2.24)	2.15 (0.68)	0.56 (1.68)	2.62 (0.84)	0.94	0.66
Support	6.07 (1.43)	6.29 (1.55)	1.77 (0.71)	–0.66 (1.04)	2.12 (0.89)	0.93	0.68
Warmth	6.26 (1.49)	6.49 (1.65)	1.76 (0.75)	–0.77 (1.67)	2.07 (0.96)	0.93	0.67
Mood	6.09 (0.97)	6.29 (1.09)	1.46 (0.58)	–0.55 (0.59)	1.75 (0.72)	0.91	0.75
Stress	4.04 (1.42)	3.81 (1.70)	1.82 (0.70)	0.37 (0.72)	2.09 (0.84)	0.93	0.68
Engagement	6.02 (1.36)	6.38 (1.71)	2.11 (0.76)	–0.59 (0.91)	2.57 (0.92)	0.9	0.74
Interaction	6.53 (1.27)	6.70 (1.54)	1.69 (0.59)	–0.56 (0.96)	2.10 (0.78)	0.93	0.64
Assertiveness	5.17 (1.20)	5.16 (1.37)	1.51 (0.61)	–0.29 (1.17)	1.94 (0.79)	0.92	0.64

Note. All items ranged from 1 (not at all) to 10 (very much).

### Dynamic exploratory graph analysis

**Fig. 2:**
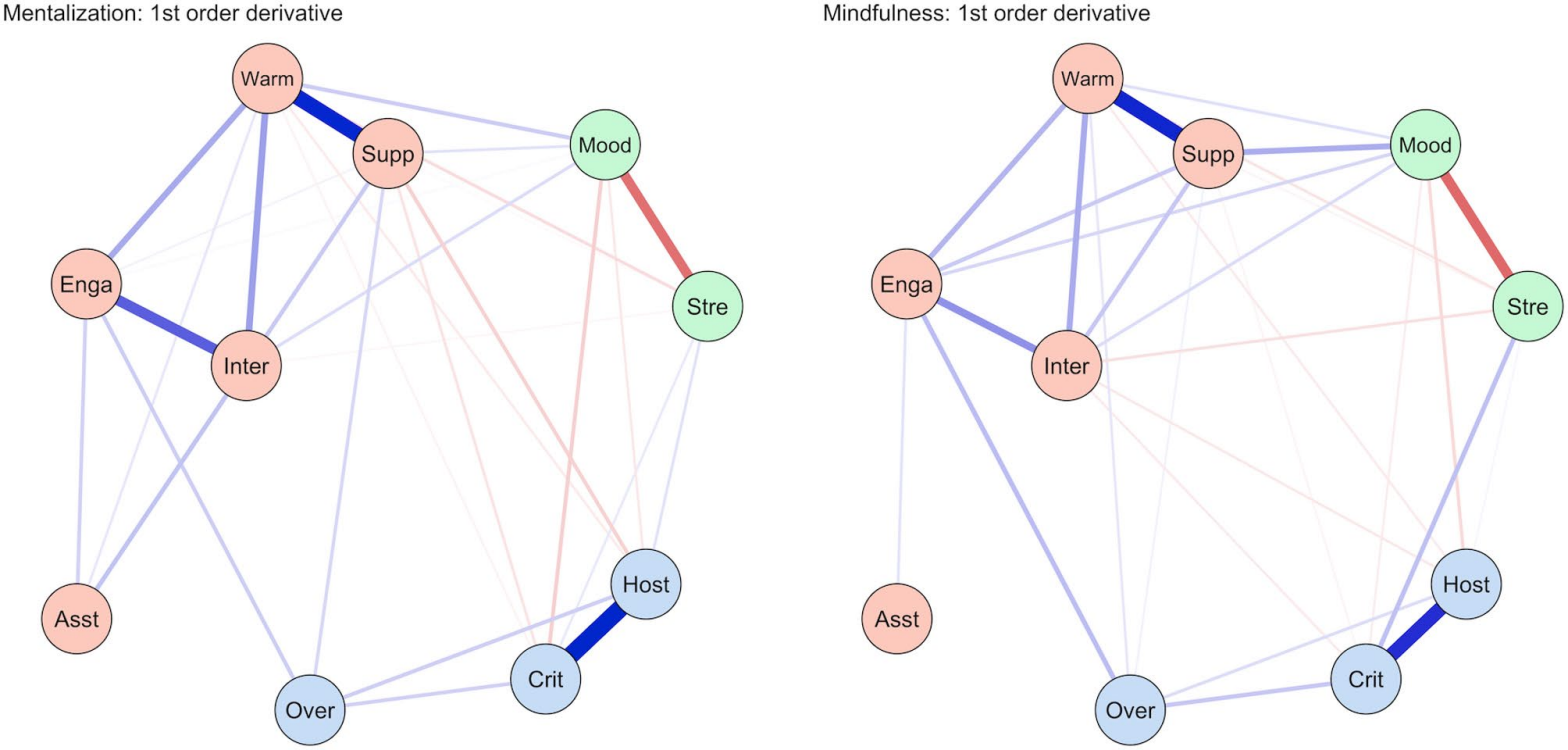
Dynamic EGA Networks Derived from EMA Data, Representing Velocity (left) and Acceleration (right) in the Mentalization and Mindfulness Conditions. Note. Each network shows the structure of dynamic relationships between ten EMA variables: warmth (warm), support (supp), engagement (enga), interaction (inter), assertiveness (asst), mood (mood), stress (stre), hostility (host), criticism (crit), and overinvolvement (over). The edges represent partial correlations between variables, indicating direct connections after controlling for all others. In the network, edges reflect velocity (i.e., the change over time). Blue edges indicate positive associations (i.e., both variables increase or decrease together), and red edges indicate negative associations (i.e., as one increases, the other decreases). Thicker edges reflect stronger connections.

**Network Similarity and Stability** Dynamic Exploratory Graph Analysis (DynEGA) was used to estimate the dynamic associations (velocity) between variables. Figure 2 visualizes these velocity networks. The global structure of symptom dynamics was highly stable across conditions, with a strong correlation between the Mentalization and Mindfulness networks in both D1: velocity (*r* = 0.94) and D2: acceleration (*r* = 0.95) dimensions. Furthermore, the correlation between velocity and acceleration networks themselves was effectively unitary (*r* = 0.99). Consequently, we report results for the velocity networks (rate of change) below.

### Communities

In both intervention arms, DynEGA identified three stable communities of co-fluctuating variables: Interpersonal Threat: Criticism, Hostility, Overinvolvement; Social Connection: Support, Warmth, Engagement, Interaction, Assertiveness; and Affective States: Mood, Stress.

### Network centrality

In both conditions, Warmth (mentalization = 1.18; mindfulness = 1.05) and Support (0.96 and 0.97 respectively) emerged as the most central nodes, indicating these dimensions exert the strongest influence on the overall network structure. Conversely, Assertiveness, Stress, and Overinvolvement consistently showed the lowest centrality across conditions. Mood emerged as the strongest bridge node between communities (mentalization 0.38; mindfulness 0.50) in both conditions, serving as the primary connector between social and emotional experiences.

### Within-Community dynamics

Strong within-community velocity connections were consistent across conditions, revealing shared patterns of change:

#### Interpersonal threat

As expected, Criticism and Hostility were strongly connected (r_partial_=0.59 in mentalization; r_partial_=0.48 in mindfulness), indicating that improvements (upwards shifts) in perceived criticism co-occurred with improvements in feelings of hostility.

*Social connection*: Intuitively, Support and Warmth demonstrated the strongest coupling (r_partial_=0.52 in both conditions). This was followed by Engagement and Interaction (mentalization: r_partial_=0.35; mindfulness: r_partial_=0.23) and Warmth and Interaction (mentalization: r_partial_=0.20; mindfulness: r_partial_=0.17).

#### Affective States

Changes in Mood showed consistent negative velocity associations with Stress (r_partial_=–0.31 in mentalization; r_partial_=–0.32 in mindfulness) confirming that mood improvements co-occurred with stress reduction.

### Intervention-Specific dynamics

Despite high global similarity, distinct edge-weight patterns emerged for each condition.

#### Mentalization-Specific patterns

Several distinct patterns of node associations emerged in the mentalization condition.

Affective States - Interpersonal Threat: Increases in Mood were uniquely associated with reductions in Criticism (r_partial_ = −0.10), suggesting a direct link between improved affect and reduced perception of interpersonal threat.

Social Connection - Interpersonal Threat: Increasing rates of Support co-occurred with decreasing rates of Criticism (r_partial_ = −0.05) and Hostility (r_partial_ = −0.09). However, Support also showed positive associations with increases in Overinvolvement (r_partial_ = 0.08). Additionally, Overinvolvement was positively associated with increases in Engagement (r_partial_ = 0.10).

Within-Community Dynamics (Social Connection): Assertiveness demonstrated unique driver effects within the social community, showing positive associations with increases in Warmth (r_partial_ = 0.05), Engagement (r_partial_ = 0.09), and Interaction (r_partial_ = 0.12).

### Mindfulness-Specific patterns

Social Connection - Affective States: The mindfulness network displayed strong connectivity between social behavior and affect. Support was positively associated with increases in Mood (r_partial_ = 0.17). This pattern was reinforced by a positive association between Engagement and increases in Mood (r_partial_ = 0.08), aligning with the finding that Mood served as a stronger bridge node in this condition.

Social Connection - Interpersonal Threat: Being warm and friendly (Interaction) showed broad inverse associations with threat and distress. Increases in Interaction co-occurred with decreases in Hostility (r_partial_ = −0.04) and Criticism (r_partial_ = −0.04). Consistent with the mentalization arm, increases in Overinvolvement co-occurred with increases in Engagement (r_partial_ = 0.13) and Warmth (r_partial_ = 0.05).

Affective States - Interpersonal Threat: Unique to the mindfulness condition, increases in Criticism were directly associated with increases in Stress (r_partial_ = 0.13). Additionally, the protective effect of social interaction extended to affect, where increases in Interaction co-occurred with decreases in Stress (r_partial_ = −0.06).

### Proximal intervention effectiveness

We examined the immediate impact of micro-interventions by comparing symptom levels at the trigger beep to levels at the subsequent assessment. The analysis included 533 paired episodes in the Mentalization arm and 636 paired episodes in the Mindfulness arm. Analysis of proximal effects (supplementary table S2) revealed no statistically significant changes across any symptom domain for either intervention condition (all *p* > 0.05). Effect sizes were negligible across all outcomes (|d| < 0.06), indicating that symptom intensity at the subsequent beep was statistically indistinguishable from intensity at the trigger moment.

The largest observed changes in the mentalization arm were non-significant reductions in *Hostility* (d = −0.048, *p* = 0.38) and *Criticism* (d = −0.042, *p* = 0.43). Similarly, the strongest observed trends in the mindfulness arm were non-significant reductions in *Hostility* (d = −0.054, *p* = 0.27) and *Criticism* (d = −0.035, *p* = 0.50).

### Predictors of intervention Non-Engagement

Analysis of 5,892 intervention trigger instances revealed a 79.3% non-engagement rate, where participants failed to interact with delivered interventions despite meeting personalized symptom thresholds. A mixed-effects logistic regression model identified six significant predictors of this non-engagement (supplementary table S3).

**Facilitators of Engagement**: The strongest predictor of intervention use was the cumulative symptom burden. For each additional symptom threshold triggered simultaneously, the odds of ignoring the intervention decreased by 31% (OR = 0.69 [95% CI: 0.62, 0.77], *p* < 0.001). This suggests that participants were significantly more likely to accept help when experiencing multiple co-occurring symptoms. Social connection also facilitated engagement; for every additional person a participant had been in contact with since the last assessment, the odds of ignoring the intervention decreased by 17% (OR = 0.83 [95% CI: 0.73–0.95], z = −2.83, *p* = 0.005). This suggests that higher volumes of recent social interaction increased the willingness to engage with an intervention.

Barriers to Engagement: For every standard deviation improvement in mood, participants were 22% more likely to ignore the prompt (OR = 1.22 [95% CI: 1.06, 1.41], *p* = 0.006), likely reflecting a perceived lack of need for intervention during positive states. Higher stress levels predicted higher non-engagement (OR = 1.21 [95% CI: 1.05, 1.40], *p* = 0.009). Both Perceived Criticism (OR = 1.22 [95% CI: 1.03–1.45], *p* = 0.026) and Emotional Overinvolvement (OR = 1.23 [95% CI: 1.05–1.44], *p* = 0.005) were significant barriers, increasing the odds of non-engagement by over 20%.

Intervention fatigue did not appear to be a factor. Neither study day progression OR = 1.04, *p* = 0.544) nor time of day (beep level; OR = 1.50, *p* = 0.390) predicted non-engagement when controlling for EMA variables.

### Dynamic prediction model performance

We developed a dynamic prediction model to forecast the risk of high distress at the next beep (t + 1) based on current symptom profiles (t). The final model included 5,233 complete observations from 73 participants.

### Model performance

The model demonstrated fair discriminative ability for predicting imminent high distress, achieving an Area Under the Curve (AUC) of 0.664 [95% CI: 0.646, 0.682]. Calibration was acceptable, with a slope of 1.15 (SE = 0.07) and an intercept of 0.20 (SE = 0.09), indicating a slight tendency to underestimate the probability of high-distress events.

### Predictors of future distress

To determine whether interpersonal factors predict *future* shifts in distress rather than just reflecting current states, the model controlled for current Mood and Stress. As expected, these autoregressive terms were the strongest predictors, indicating substantial state persistence (supplementary table S4).

Each SD increase in current stress increased the odds of next-beep distress by 37% (OR = 1.37, 95% CI [1.26, 1.50], *p* < 0.001). Higher current mood was protective, with each SD increase associated with a 21% reduction in distress odds (OR = 0.79, 95% CI [0.72, 0.87], *p* < 0.001).

Risk Factors: After adjusting for baseline mood and stress, specific interpersonal context variables were significant independent risk factors for next-beep distress. Unexpectedly, higher perceived support was associated with a 14% increase in the odds of subsequent distress (OR = 1.14, 95% CI [1.00, 1.31], *p* = 0.045). Higher criticism was an independent risk factor, associated with a 12% increase in distress odds (OR = 1.12, 95% CI [1.00, 1.25], *p* = 0.046).

Protective Factors: Higher warmth (perceived warmth from others) showed a significant protective effect (OR = 0.87, 95% CI [0.76, 0.99], *p* = 0.032). Distress risk significantly decreased as the day progressed. Each additional beep was associated with an 11% reduction in distress odds (OR = 0.89, 95% CI [0.83, 0.96], *p* = 0.002).

Neither the cumulative number of triggers (OR = 0.94 [0.87, 1.01], *p* = 0.074) nor the receipt of a recent successful intervention (OR = 0.85 [0.69, 1.06], *p* = 0.141) significantly predicted the *next* distress state after controlling for current mood and stress, suggesting these factors may not exert a lagged protective effect beyond their immediate impact on the current state.

#### Sensitivity analysis: continuous prediction of distress

To evaluate the robustness of the predictive model, a sensitivity analysis was conducted using a Linear Mixed Model with a continuous distress score as the outcome (supplementary table S5). The results were largely consistent with the primary binomial (GLMM) analysis. Current affective states remained the strongest predictors of future status; higher current stress predicted increased future distress (B = −0.26, *p* < 0.001), while better current mood predicted lower future distress (B = 0.17, *p* < 0.001).

Perceived Criticism remained a significant risk factor (B = −0.09, *p* < 0.001), whereas Warmth exerted a significant protective effect (B = 0.07, *p* = 0.024). Unlike the binomial model, the linear model identified a higher cumulative number of triggers (B = 0.07, *p* < 0.001) and weekends (B = 0.09, *p* = 0.009) as associated with reduced subsequent distress. Additionally, a significant effect of study day (B = 0.06, *p* = 0.011) indicated a general trend of symptom improvement over the course of the study. The model explained a small portion of the variance (Marginal R2 = 0.12).

## Discussion

The objective of this study was to examine the mechanisms of change, predictive dynamics and optimization challenges of mindfulness and mentalization micro-interventions for interpersonal distress in adults with CMDs. While the parent trial demonstrated significant distal symptom reduction^27^, this secondary analysis revealed a picture of *how* and *when* these changes unfold. The dynamic network analysis revealed distinct mechanisms of change, yet there were no significant proximal effect patterns. Non-engagement patterns revealed that the very states necessitating intervention (high stress, interpersonal threat) act as barriers to utilizing it. A dynamic prediction model predicting next-beep distress showed that, while immediate emotional states (mood and stress) were the primary predictors of short-term distress, specific interpersonal context variables and temporal context acted as independent predictors. This discussion synthesises these findings to inform future EMI approaches that must balance therapeutic effectiveness with sustainable engagement strategies.

The discrepancy between the significant distal improvements observed in the parent trial and the absence of significant proximal (next beep) effects in this analysis suggests that mindfulness and mentalization micro-interventions do not function as immediate remedies, but rather through cumulative exposure. However, it may also reflect the influence of common factors or expectancy effects (placebo) inherent in the trial’s design. Proximal outcomes theoretically link the adaptation process to the distal outcome^41^. However, we found that successful intervention delivery did not reliably predict symptom improvement at the subsequent beep (approx. 2 h later). This implies that the therapeutic dose of a single micro-intervention may be insufficient to shift the inertia of established mood states immediately. Instead, benefits likely accrue through repeated practice, gradually altering the baseline network structure over days or weeks. Alternatively, the null proximal result may be an artifact of the measurement window: intervention may miss the optimal state for delivery, and when these ineffective intervention instances are averaged with the few, possibly effective ones, the overall result is a statistically small and insignificant finding^21^, or emotional shifts may occur on a faster timescale than our sampling interval captured^42^. Future designs should employ flexible-lag assessments to capture rapid affect dynamics.

### Intervention mechanisms

Dynamic network analysis (DynEGA) elucidated *how* these interventions operate. Consistent with common factors theory^43^, which posits that distinct therapeutic approaches operate through shared mechanisms, both interventions shared a stable three-community structure (Interpersonal Threat, Social Connection, Affective States), with Mood serving as the primary bridge node. This supports transdiagnostic models emphasizing affect regulation as a core process across evidence-based interventions^44^.

However, unique velocity patterns revealed distinct mechanistic signatures. In the mentalization arm, co-occurring rate of change between mood improvements and criticism reduction, along with stronger links connecting assertiveness to both receiving and expressing warmth, and to the support-overinvolvement and support-hostility dyads suggests mentalizing operates by restoring interpersonal cognitive clarity. By enhancing the capacity to distinguish self-states from others’ intentions, individuals may reduce the projection of distress onto their social environment^45^. The stronger assertiveness-warmth connections in mentalizing may reflect increased confidence in expressing needs when interpersonal perceptions become more accurate and less contaminated by affective states.

In contrast, mindfulness showed strong connectivity between Support, Engagement, and Mood. Uniquely, increases in Interaction (friendliness) co-occurred with decreases in Stress and Hostility. Critically, mindfulness was also associated with increased co-occurrence of Criticism-Stress and Engagement-Overinvolvement. Rather than deterioration, this likely reflects enhanced awareness: an improved capacity to detect interpersonal threats and boundary intrusions (overinvolvement) without avoidance^46^. This interpretation aligns with research demonstrating that mindfulness increases tolerance for negative affective experiences while reducing reactivity^47,48^. The protective warmth patterns suggest that mindfulness may buffer against interpersonal stress by enhancing behavioral flexibility and prosocial responding even under challenging conditions.

### Engagement challenges and predictors

Despite not being perceived as burdensome^27^, micro-interventions were frequently ignored. Participants were significantly less likely to engage when experiencing high Stress, Perceived Criticism, or Emotional Overinvolvement. This aligns with the Yerkes-Dodson Law^49^ and research on stress-induced executive dysfunction, working memory, and deliberate self-regulation^50,51^. Acute arousal likely impairs the cognitive bandwidth required to initiate self-regulation, rendering the intervention inaccessible precisely when it is most needed. Criticism, in particular, may undermine the self-efficacy required for engagement^52,53^.

Conversely, “Total Triggers” (cumulative symptom load) facilitated engagement. This suggests that while specific acute stressors (e.g., criticism) trigger avoidance, a broad “multi-symptom” crisis may overwhelm avoidance and trigger help-seeking. Additionally, social contact facilitated engagement. Broader social engagement, despite occurring in the context of elevated interpersonal stressors, may provide opportunities for social support that buffer against these stressors through enhanced relatedness (a sense of being connected and understood) or through interpersonal reappraisal processes^52,54^. Alternatively, being with others may increase accountability or reduce competing demands on attention, making intervention engagement more feasible^55,56^.

During elevated mood, participants may perceive less immediate need for intervention, leading to avoidance despite the presence of other elevated symptoms that triggered the intervention. This creates a distinct but equally challenging engagement barrier: individuals dismiss therapeutic content when feeling affectively well, even when interpersonal or stress indicators suggest vulnerability. The bridging role of mood within the dynamic network may explain why mood emerged as a significant predictor in both distress prediction and non-engagement models: mood fluctuations cascade across the entire symptom network, influencing both future distress risk and intervention receptivity through its connections to interpersonal threat and social connection communities.

Viewing these predictive patterns jointly reveals that engagement was most likely not at the extremes of experience, but rather in a state of ‘manageable distress.’ While positive mood likely reduced the perceived relevance of the intervention (low need), high stress and interpersonal threat appeared to overwhelm the cognitive capacity required to use it (low capacity). This implies a distinct window of receptivity for micro-interventions, a zone where symptoms are sufficiently elevated to motivate help-seeking but not so acute that they paralyze action. Consequently, future JITAI algorithms may need to move beyond binary thresholds to a stepped approach: confirming need during positive states to reduce fatigue, and pivoting to lower-friction, simplified support during peaks of acute arousal.

### Dynamic prediction

Current stress and mood emerged as the strongest predictors of subsequent distress, confirming the phenomenon of state persistence (inertia) in high-risk samples. Higher stress predicted continued distress, while positive mood facilitated a regression to the mean (recovery). After controlling for current emotional states, interpersonal context variables showed significant but divergent predictive utility, challenging the view that all positive relationship variables function similarly.

Criticism independently predicted increases in next-beep distress. Criticism showed consistent patterns: meaningful predictor of subsequent distress and increased intervention avoidance. This aligns with research identifying perceived criticism as uniquely predicting relapse and treatment disengagement^53^, suggesting that criticism detection might trigger modified content addressing self-efficacy and hope. Counter-intuitively, higher perceived support predicted an increase in subsequent distress odds. Rather than signalling a failure of support, this likely reflects reactive mobilization (seeking support because one is becoming distressed) or co-rumination (where discussing problems intensifies the focus on distress without resolving it). In contrast to support, perceived warmth functioned as a significant protective factor. While Support is transactional, Warmth represents a relational climate of acceptance. This suggests that interventions should aim to foster the perception of warmth (safety) rather than merely encouraging support-seeking behaviours, which may inadvertently reinforce distress focus^57^.

Temporal factors played a significant protective role, with distress risk decreasing as the day progressed. This diurnal pattern suggests that risk is highest earlier in the day - potentially due to morning anticipation of stressors or cortisol awakening responses - and tends to attenuate as the day resolves.

To translate these predictive findings into actionable decision-making guidance, they could be developed into a simplified point-based risk score derived from the model’s strongest predictors. However, this framework would require prospective validation in an independent sample, particularly to assess whether risk-stratified intervention delivery achieves comparable clinical outcomes with reduced participant burden.

### Limitations

The high non-engagement rate may have created selection effects, limiting interpretation of proximal effectiveness analyses. Dynamic network analysis lacks temporal directionality, and as such we could not identify where change in one symptom predicted change in another. Additionally, some participants had fewer than recommended time points for stable estimation^58^. The sample was primarily university students, limiting generalisability to broader clinical populations. Different cultural backgrounds may also influence interpretations of interpersonal behaviours such as overinvolvement^59,60^, requiring examination in future research.

JITAI delivery is triggered by symptom states that simultaneously drive outcomes, and engagement further modifies subsequent trajectories - a form of treatment-confounder feedback where past treatment affects future confounders and outcomes^61^. As a result, network patterns, proximal effects and prediction results may reflect endogenous dynamics rather than causal effects. Sensitivity analyses across alternative trigger frameworks are needed to assess generality.

### Implications

This study demonstrates the value of methodological triangulation in providing insights that individual approaches would miss. For example, stress emerged consistently as the strongest predictor of future distress yet simultaneously acted as the primary barrier to engagement. This was only visible by integrating predictive and engagement frameworks. A purely predictive model would suggest triggering interventions during high stress, while an engagement model would warn against it. This validates the necessity of multi-method approaches in digital health to identify not just when an intervention is needed, but whether it can be received^21^.

Our findings extend Expressed Emotion theory by demonstrating that interventions targeting individual capacities can alter interpersonal perception without requiring family-level intervention. Specifically, the nuances of Emotional Overinvolvement warrant re-conceptualization. While Overinvolvement co-occurred with criticism and hostility (supporting traditional interpretations of intrusive control)^62^, it also co-occurred with engagement, support and warmth. This suggests that participants may have increasingly interpreted others’ involvement as responsive care rather than intrusive control^63–65^. Alternatively, the intervention may have enhanced the participant’s capacity to distinguish genuine concern from criticism, reducing an attributional bias^66^. This implies that Overinvolvement is a dynamic construct whose impact depends on the concurrent presence of warmth versus hostility.

The high non-engagement rates with the intervention prompts observed do not indicate intervention failure but rather reveal optimal intervention windows and readiness factors requiring design adaptation. Participants demonstrated high overall adherence and acceptability in the trial, suggesting they were willing to engage with the intervention generally, but selectively filtered specific prompts. The current algorithm (triggered at 1 SD above the rolling average) frequently deployed interventions during minor fluctuations or even when participants were in positive mood states, a significant predictor of ignoring the prompt. This indicates the system often attempted to intervene when help was not subjectively required. To align clinical need with user receptivity, future JITAI designs require adaptations. Algorithms must distinguish between high and moderate arousal states. During high stress (where cognitive bandwidth is low), simplified interventions could be deployed (e.g., grounding exercises), or cognitively demanding reflective exercises postponed. To sustain engagement over clinically meaningful timeframes, future trials could test adaptive measurement schedules, such as “front-loaded” intensive assessment periods^67^ followed by maintenance phases.

## Conclusion

This study demonstrates that micro-interventions can achieve theoretically coherent, targeted effects through distinct mechanisms while revealing fundamental challenges in sustaining engagement over intensive protocols. Using predictive models, we can optimise these approaches and advance toward precision mental health systems. By holding engagement as a co-primary endpoint alongside clinical outcomes, future approaches can navigate the tension between therapeutic intensity and sustainable participation.

## Supplementary Information

Below is the link to the electronic supplementary material.

Supplementary Material 1

## Data Availability

The datasets generated or analyzed during this study are available in the Open Science Framework (OSF) repository: [https://osf.io/chfy2/].
