# Supplementary material for: Optimizing just-in-time adaptive interventions for interpersonal distress: mechanisms, prediction, and the challenge of engagement
Source: Sci Rep. 2026 Feb 11;16:8406. doi: 10.1038/s41598-026-39518-z (PMC12972048; doi:10.1038/s41598-026-39518-z)
Supplement: Supplementary file 1 — Supplementary Material 1 [file 41598_2026_39518_MOESM1_ESM.docx]

**Supplementary Materials:**

**Table S1. EMA items**

| **Construct** | **Question** | **Slider (0 to 10)** |
| --- | --- | --- |
| Mood | How do you rate your mood since the last beep? | Smiley:valence |
| Stress | How stressed do you feel? | Smiley:stress |
| Criticism | Since the last beep I have felt criticized | Anchors (Not at all / Very) |
| Hostility | Since the last beep I have experienced hostility | Anchors (Not at all / Alot) |
| Overinvolvement | Others are insisting on being involved with what I am doing | Anchors (Not at all / Alot) |
| Support | I feel highly supported | Anchors (Not at all / Alot) |
| Warmth | I feel valued or close to others | Anchors (Not at all / Very) |
| Engagement | I have been engaging with activities / others | Anchors Avoiding to Engaging) |
| Interaction | In my interactions I have been: | Anchors: Cold/Hostile to Warm/Friendly) |
| Assertiveness | In my interactions I have been: | Anchors: Submissive to Assertive/Dominant) |

**Figure S1**

**
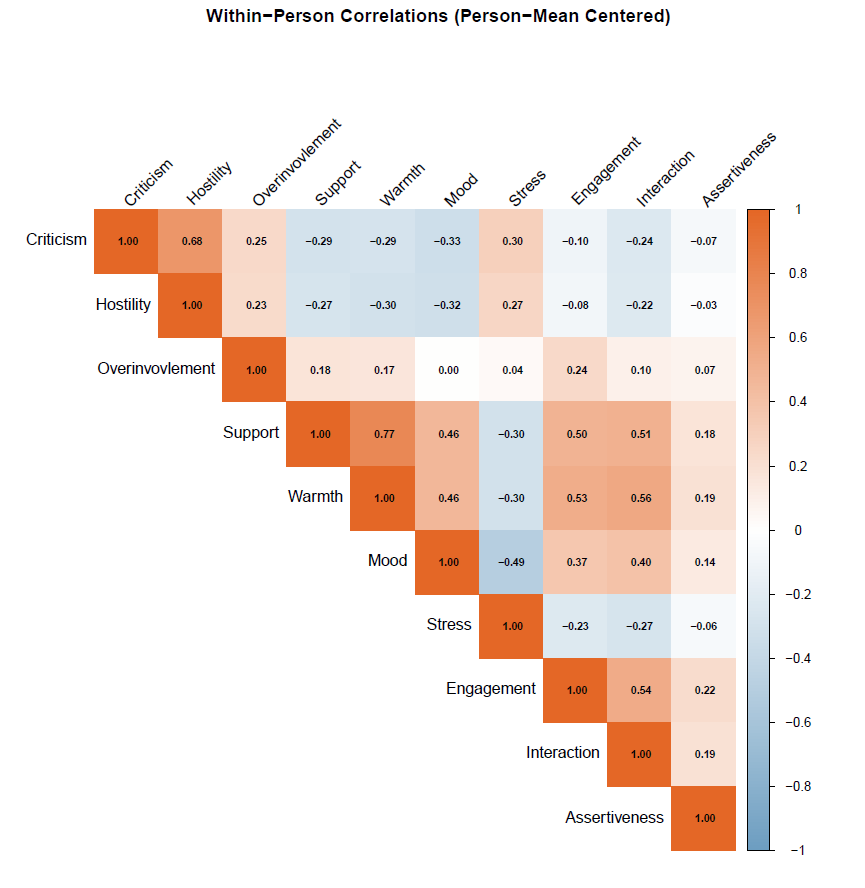
**

**Proximal Effects of Interventions**

**Table S2. Fixed Effects of Mentalization and Mindfulness Interventions on Immediate Outcome Measures**

| **Intervention Group** | **Outcome Measure** | **n (pairs)** | **Cohen's d** | **95% CI** | **p** |
| --- | --- | --- | --- | --- | --- |
| Mentalization | Hostility | 533 | -0.048 | [-0.153, 0.058] | .376 |
|  | Criticism | 533 | -0.042 | [-0.148, 0.063] | .431 |
|  | Mood | 533 | 0.040 | [-0.066, 0.147] | .458 |
|  | Assertiveness | 528 | -0.037 | [-0.124, 0.049] | .399 |
|  | Support | 533 | 0.028 | [-0.068, 0.124] | .570 |
|  | Interaction | 528 | 0.025 | [-0.064, 0.114] | .581 |
|  | Overinvolvement | 533 | -0.020 | [-0.108, 0.068] | .659 |
|  | Stress | 533 | 0.016 | [-0.077, 0.108] | .741 |
|  | Engagement | 528 | -0.007 | [-0.099, 0.086] | .888 |
|  | Warmth | 533 | -0.001 | [-0.101, 0.099] | .987 |
| Mindfulness | Hostility | 636 | -0.054 | [-0.150, 0.042] | .272 |
|  | Criticism | 636 | -0.035 | [-0.136, 0.066] | .497 |
|  | Stress | 636 | -0.021 | [-0.131, 0.090] | .712 |
|  | Overinvolvement | 636 | 0.020 | [-0.076, 0.116] | .685 |
|  | Mood | 636 | 0.017 | [-0.080, 0.115] | .726 |
|  | Assertiveness | 631 | -0.017 | [-0.104, 0.069] | .697 |
|  | Warmth | 636 | -0.011 | [-0.107, 0.084] | .814 |
|  | Support | 636 | -0.002 | [-0.105, 0.101] | .969 |
|  | Engagement | 631 | 0.001 | [-0.097, 0.098] | .991 |
|  | Interaction | 631 | 0.000 | [-0.088, 0.088] | >.999 |

**Non-Engagement Analysis**

**Table S3: Fixed Effects Predictors of Non-Engagement**

| **Predictor** | **B** | **SE** | **OR** | **95% CI** | **p** |
| --- | --- | --- | --- | --- | --- |
| Intercept | 2.94 | 0.44 | 18.94 | [7.98, 44.98] | < .001 |
| **Psychological State** |  |  |  |  |  |
| Mood | 0.20 | 0.07 | 1.22 | [1.06, 1.41] | .006 |
| Stress | 0.19 | 0.07 | 1.21 | [1.05, 1.40] | .009 |
| Criticism | 0.20 | 0.09 | 1.22 | [1.02, 1.46] | .026 |
| Overinvolvement | 0.21 | 0.07 | 1.23 | [1.06, 1.42] | .005 |
| Support | 0.19 | 0.10 | 1.21 | [1.00, 1.48] | .053 |
| Hostility | -0.11 | 0.09 | 0.90 | [0.75, 1.07] | .239 |
| Warmth | -0.12 | 0.11 | 0.89 | [0.72, 1.10] | .272 |
| Engagement | -0.01 | 0.08 | 0.99 | [0.86, 1.16] | .937 |
| Interaction Quality | 0.04 | 0.08 | 1.05 | [0.89, 1.23] | .579 |
| Assertiveness | -0.04 | 0.06 | 0.96 | [0.85, 1.09] | .525 |
| **Context & Intervention** |  |  |  |  |  |
| Triggers Exceeded | -0.38 | 0.06 | 0.69 | [0.62, 0.76] | < .001 |
| Social Contact | -0.18 | 0.06 | 0.83 | [0.73, 0.95] | .005 |
| Intervention Count | 0.56 | 0.45 | 1.74 | [0.72, 4.24] | .220 |
| Beep Number | 0.38 | 0.44 | 1.46 | [0.61, 3.47] | .392 |
| Study Day | 0.04 | 0.06 | 1.04 | [0.92, 1.17] | .544 |
| Weekend (vs. Weekday) | -0.03 | 0.11 | 0.97 | [0.78, 1.20] | .793 |
| Group (Mindfulness) | -0.28 | 0.61 | 0.76 | [0.23, 2.51] | .646 |

**Dynamic Prediction Model**

**Table S4. Fixed Effects Predictors of Next-Moment High Distress (Binary - GLMM)**

| **Predictor** | **B** | **SE** | **OR** | **95% CI** | **p** |
| --- | --- | --- | --- | --- | --- |
| **Psychological State** |  |  |  |  |  |
| Stress | 0.32 | 0.04 | 1.37 | [1.26, 1.50] | < .001 |
| Mood | -0.23 | 0.05 | 0.79 | [0.72, 0.87] | < .001 |
| **Interpersonal** |  |  |  |  |  |
| Support | 0.14 | 0.07 | 1.14 | [1.00, 1.31] | .045 |
| Criticism | 0.11 | 0.06 | 1.12 | [1.00, 1.25] | .046 |
| Warmth | -0.14 | 0.07 | 0.87 | [0.76, 0.99] | .032 |
| Overinvolvement | 0.02 | 0.04 | 1.02 | [0.93, 1.11] | .722 |
| Hostility | -0.01 | 0.05 | 0.99 | [0.89, 1.10] | .798 |
| **Context & History** |  |  |  |  |  |
| Beep (Time of Day) | -0.11 | 0.04 | 0.89 | [0.83, 0.96] | .002 |
| Total Triggers (Cumulative) | -0.07 | 0.04 | 0.94 | [0.87, 1.01] | .074 |
| Successful Recent Intervention | -0.16 | 0.11 | 0.85 | [0.69, 1.06] | .141 |
| Weekend | -0.11 | 0.08 | 0.89 | [0.76, 1.04] | .144 |
| Study Day | -0.02 | 0.04 | 0.98 | [0.90, 1.06] | .625 |
| Contact (Social) | -0.01 | 0.04 | 0.99 | [0.91, 1.08] | .848 |

**Table S5. Sensitivity Analysis: Fixed Effects Predictors of Next-Moment Distress (Linear - LMM)**

| **Predictor** | **B** | **SE** | **95% CI** | **p** | **Cohens d** |
| --- | --- | --- | --- | --- | --- |
| Stress | -0.26 | 0.022 | [-0.303, -0.217] | <0.001 | -0.217 |
| Mood | 0.168 | 0.023 | [0.123, 0.213] | <0.001 | 0.14 |
| Criticism | -0.089 | 0.027 | [-0.142, -0.036] | <0.001 | -0.074 |
| Warmth | 0.072 | 0.032 | [0.009, 0.135] | 0.024 | 0.06 |
| Support | -0.035 | 0.031 | [-0.097, 0.026] | 0.259 | -0.03 |
| Overinvolvement | -0.011 | 0.021 | [-0.053, 0.030] | 0.593 | -0.009 |
| Hostility | 0.01 | 0.027 | [-0.042, 0.062] | 0.709 | 0.008 |
| Total Triggers (count) | 0.065 | 0.017 | [0.031, 0.098] | <0.001 | 0.054 |
| Weekend | 0.09 | 0.035 | [0.022, 0.159] | 0.009 | 0.076 |
| Study Day | 0.055 | 0.022 | [0.013, 0.098] | 0.011 | 0.046 |
| Time of Day | 0.001 | 0.016 | [-0.031, 0.033] | 0.964 | 0.001 |
| Recent Successful Intervention | 0.033 | 0.051 | [-0.066, 0.132] | 0.515 | 0.028 |
| Social Contact (count) | 0.031 | 0.021 | [-0.010, 0.073] | 0.142 | 0.026 |

**Note:** Marginal R² =0.123, Conditional R² = 0.152, Intraclass Correlation = 0.033
